# Monthly Record of Deaths in the City of Buffalo

**Published:** 1855-05

**Authors:** J. Root

**Affiliations:** Health Physician


					﻿ART. IV. — Report of Deaths in Buffalo for the month
AGE.
-J	cZ
i- O
- k. c
P - A H;
DISEASES.	.	----•----
a
1	. o	.	.
S	3	05 1 « 2
* * I £ s I
Apoplexy,..........................-.................. 1 ----	1	-- -...
Anaemia,..................-................................ 1	1	.......
Bronchitis,................................■.......... 3	1	4	2......
Consumption,..................-....................... 9	9	15 | 1 .. .....
Convulsions,..........-.......-....................... 8	8	16	4 4 2 3
Child Bed,............................................2	2  ........
Croup, ........................... —	............... 4	4	8	2 1 .. 1
Canker,.................................................... 1	1 -- 1--..
Congestion of Brain,....................................... 1	1 .........
“	“ Lungs,....................................... 1	1	...........
Dysentery,............................................ 2	1	3	..........
Diarrhoea,................................................. 1	1	....... 1
Debility,........................................... -	1	4	5	14;...
Dropsy, —.................................................. 1	1 j............
Dentition,............................................ 1	----	1	•	1..
Erysipelas,................................................ 1	1	..........
Fever, Typhus,........................................ 3 ....	3	..........
“ Typhoid,....................  ................... 1	....	1	...........
“ Scarlet,......................................... 3 ....	3	...........
Haemorrhage,............................................... 1	1	..........
Heart Disease,........................................ 1	1	2	..........
Inflammation of Brain,.....................-.........-	1	-—	1	1 .. .. ..
**	“ Bowels,................................ 2 ....	2	...........
Intestinal Irritation,................................ 1	....	1	1 -. .. ..
Jaundice,..........-...............—	—.............. 1	■—	1	.........
Marasmus, -...............-................-.......... I 1	2	1,1....
Measles,..............................................—■- 1 1
Old Age,...................................-.......... 3 ------ 3	.......... .
Paralysis,............................................).....	1	1 - - - - {- - ..
Pneumonia,............................................ 5	8	13	1 1 .. 3
Phlibitis,............................................ 1 ----	1	..........
Suicide,.......................--................... —	2 .—	2	..........
Spine Disease,........................................ 1	-—	1	...........
Still-born,........................................... 1	1	2 ...........
Tracheitis,........................................... 1	----	1	..........
Unknown,.............................................. (> •’ 12 2 4 1 __
"Whooping Coughy........................-............. 1	3	4	.. 2 J j
Totals,..............................~64~ ~59~ I23~ 16'TU~5 9
Nativities.—American,42. German,43. English,5. Irish, 12. Scotch, 1. Swiss>2.
Country not given, 8.—-Total, 123.
of March, 1855. By J. Root, M. D., Health Physician.
AGE.
I-I	•	§
m	o	'	irt	o	a	o	o	o	o	o	o i	x	?
io	I	o	r-i	ci	Cl	co	■d’	m	w	<-	ao	co	cd	■ -	j-
I	.	7	I	I	'	I	i	I	I	i	I	I	I	7	o	£	“
Cl	I OiO O^OOOOOO	I o P-
uQ	Qi Qt CQ	GC L- GO O
________________________I_________________________"	~
C _ci	' ci _ci	i ci	ci si _ci I ci ci ci ci ci ci ci
_x r	j r	_x -	s r	- ~	r	s §	jc r	s ~	a c	as J I	aS g	_re c	J; 5	~ ,Q
------------------------—------------------------------------.
.................................... 1......................
................................................................................................................................ .... 1.....
............................................................................................................................................ 1. J.........................................................................................................................................................................................................................................................................
.... 1 .. .. 1 .. .. ] 2 1 1 2 4-- 1 2 .. 1..................
1	1 1...................L.......I........................
.. ........... 1............ 1.....i.........................
1 2 1............................'.........................
.... i...................::::........  ,.....................
.............................................i............
.. i...........i..........   i...............................
------................ i--...................................
■■ i ::::'.’. ’■:::: :: :::::: :: :: :: :: :t: .: :::: :t: :::: ::::::::::
.............. i............................................
................................................................ i..............................................................!.
............................................................................................................................................ i.. i...... i.............................................................................................................................................................................................................................................................
...................................................................................................................................................................................................................................................................................................................................................................................................................... i...
2	.. 1....................................................
........................ 1...................................
............. 1 .. .. 1......................................
:::: ’i":: ’:::::::::: l|" " ::p::::::::p" ■::	::
:::: ::p:::::::: ::::	:::::::::: t :::::: t ::::::::p ::
.........................  i.............
......................................... 1. 1. 1 2 .. 1. 1.
1.................. 1 .....................................
...................................... 1 .. 1...............
............................................................. 1.
| | ......................................
i::.........................t..	:... i.....................i
i.............................................................
.............................................................
........................
.............................................................
............................................................. *■
:::::::.................::::::..:::.:...::...................
:.:::::::::::.::4:::::::..:::::: " "p " ” :: :: " :: " " :: "
........................
.......................................t	: t::::::: ::::: :: •
:::::::::::::::: ...:::::::::: :: .. 	..................
6~7~4~2~2 6 5~~0 0 ~1 2 1 0 0 0 0^ 0 1
French, 4. Prussian,!. Canadian,!. West Indian, 1. Indian,!. Colored, 2.
				

